# Diagnostic Allele-Specific PCR for the Identification of *Candida auris* Clades

**DOI:** 10.3390/jof7090754

**Published:** 2021-09-13

**Authors:** Hans Carolus, Stef Jacobs, Celia Lobo Romero, Quinten Deparis, Christina A. Cuomo, Jacques F. Meis, Patrick Van Dijck

**Affiliations:** 1 Laboratory of Molecular Cell Biology, Department of Biology, Institute of Botany and Microbiology, KU Leuven, 3001 Leuven, Belgium; hans.carolus@kuleuven.be (H.C.); stef.jacobs@student.kuleuven.be (S.J.); celia.loboromero@kuleuven.be (C.L.R.); 2 VIB-KU Leuven Center for Microbiology, 3001 Leuven, Belgium; quinten.deparis@kuleuven.be; 3 Laboratory for Genetics and Genomics, Centre for Microbial and Plant Genetics, KU Leuven, 3001 Leuven, Belgium; 4 Broad Institute of MIT and Harvard, Cambridge, MA 02142, USA; cuomo@broadinstitute.org; 5 Department of Medical Microbiology and Infectious Diseases, Canisius-Wilhelmina Hospital, 6532 Nijmegen, The Netherlands; j.meis@cwz.nl; 6 Centre of Expertise in Mycology Radboudumc/CWZ, 6532 Nijmegen, The Netherlands

**Keywords:** *Candida auris*, diagnostics, clades, PCR

## Abstract

*Candida auris* is an opportunistic pathogenic yeast that emerged worldwide during the past decade. This fungal pathogen poses a significant public health threat due to common multidrug resistance (MDR), alarming hospital outbreaks, and frequent misidentification. Genomic analyses have identified five distinct clades that are linked to five geographic areas of origin and characterized by differences in several phenotypic traits such as virulence and drug resistance. Typing of *C. auris* strains and the identification of clades can be a powerful tool in molecular epidemiology and might be of clinical importance by estimating outbreak and MDR potential. As *C. auris* has caused global outbreaks, including in low-income countries, typing *C. auris* strains quickly and inexpensively is highly valuable. We report five allele-specific polymerase chain reaction (AS-PCR) assays for the identification of *C. auris* and each of the five described clades of *C. auris* based on conserved mutations in the internal transcribed spacer (ITS) rDNA region and a clade-specific gene cluster. This PCR method provides a fast, cheap, sequencing-free diagnostic tool for the identification of *C. auris*, *C. auris* clades, and potentially, the discovery of new clades.

## 1. Introduction

*Candida auris* was first described in 2009 [1] and has emerged on three different continents in less than a decade [2]. *C. auris* is closely related to *Candida haemulonii*, *Candida duobushaemulonii*, and *Candida pseudohaemulonii*, forming a group of emergent, multidrug resistant (MDR) *Candida* species that are distantly related to *C. albicans* and *C. glabrata* [3]. Misidentification of *C. auris* is an alarming and frequently reported problem [4,5,6]. The commonly used phenotypic/biochemical identification platforms often fail to identify *C. auris* correctly. Traditional kits such as Vitek 2 YST or API 20C often lead to the misidentification of *C. auris* as *C. haemulonii, C. duobushaemulonii*, *C. sake, C. famata, C. guilliermondii, C. lusitaniae, C. parapsilosis, C. intermedia, C. catenulate, Saccharomyces kluyveri, Saccharomyces cerevisiae, Rhodotorula glutinis*, or *Rhodotorula rubra* [4,7].

Currently, the most reliable, efficient, and therefore, recommended methods for *C. auris* identification are matrix-assisted laser desorption ionization time-of-flight mass spectrometry (MALDI-TOF MS) and rDNA sequencing [4,8]. The disadvantage of these methods is the cost, high-tech equipment, and skilled labor they require. Recently, several PCR and qPCR assays were designed for rapid, cheap, and accurate identification of *C. auris* [4,9,10,11].

Whole-genome sequencing of *C. auris* isolates has shown that strains from different continents group into five distinct clades associated with a primary geographical distribution: the South Asian clade or ‘clade I’, the East Asian clade or ‘clade II’, the South African clade or ‘clade III’, the South American clade or ‘clade IV’, and the Iranian clade or ‘clade V’ [12,13]. All *C. auris* strains, isolated from patients in at least 47 countries, cluster in one of those five clades [14,15]. While the within-clade diversity is limited, thousands of single-nucleotide polymorphisms (SNPs) differentiate the five different clades, sufficient to extrapolate that hundreds of years of divergent evolution separates these genotypes [2]. Different clades have been associated with significantly divergent karyotypes, and mating type loci are clade-specific [3]. Moreover, different clades show clade-specific signatures of selection regarding cell surface manno-proteins [16] and drug-related genes [17]. Overall, this vast genomic diversity between clades translates into clade-specific phenotypes [2,3]. Clade II harbors the greatest percentage of drug-susceptible isolates [2], while clade I has the greatest reported percentage of MDR and pan-resistant isolates and is commonly resistant to fluconazole [2,13,18] and amphotericin B [2,13]. Clade III isolates are most often resistant to fluconazole, but resistance to amphotericin B is rare [2,19]. Clade IV isolates have the highest percentage of echinocandin resistance, which only sporadically occurs in clades I and III [2]. Moreover, specific drug resistance-associated mutations have been associated with specific clades [2]. Clade II and V are not associated with hospital outbreaks and commonly cause ear infections, while fungemia is rare [12,17,20,21,22]. Other studies suggest that clade II isolates are more susceptible to disinfection methods [23,24,25]. Isolates from clade I, III, and IV are, on the other hand, responsible for major hospital outbreaks of frequently multidrug resistant and lethal candidemia [17]. Isolates of clade I, III, and IV are more effective at colonization and dissemination from the gastrointestinal tract, compared to clade II strains in mice [26], while clade I and IV yield higher mortality rates compared to clade II and III in a murine systemic infection model [27]. Another important characteristic with regard to colonization, virulence, persistence in the environment, and thus, nosocomial transmission is biofilm formation [28,29], which also varies significantly between the clades [26,30].

To identify to which clade a strain of *C. auris* belongs, whole-genome sequencing (WGS) is the gold-standard approach [2,3,12,13,17]. Several other methods for typing *C. auris* strains have been investigated, including microsatellite typing, ITS sequencing, AFLP (amplified fragment length polymorphism) fingerprinting, MALDI-TOF MS, and Fourier-transform infrared spectroscopy [31]. Nevertheless, only microsatellite typing and STR (short tandem repeat) typing has been proven to be a reliable alternative to WGS for *C. auris* clade identification [31,32]. Although ITS sequencing was reliable to differentiate three out of four clades, it showed less resolution compared to microsatellite typing, as *C. auris* clade I and clade III show no variation within the ITS region [31]. Typing of *C. auris* clades has a role in molecular epidemiology, allowing tracing the origins of nosocomial transmission [2,19,32,33], while this might also confer a clinical value, as it can help to estimate virulence, transmission, and resistance tendencies [2,33].

AS-PCR can be used to verify specific single-nucleotide polymorphisms (SNPs) and deletions or insertions (indels). By designing an allele-specific variation at the 3’ end of a PCR primer and incorporating mismatches, amplification is only successful for that allele at a certain (elevated) primer annealing temperature (Ta) range [34]. In doing so, the presence of a specific allele can be screened for reliably, rapidly, cost-efficiently, and without sequencing by AS-PCR. AS-PCR can be used to identify mutations in research [35,36], or as a diagnostic tool [37,38,39,40].

In this study, we investigated specific SNPs, indels, and molecular markers to reliably identify *C. auris* and the five clades currently described. We optimized a straightforward, cheap, fast, and easy-to-interpret PCR assay that relies on allele-specific amplification to identify *C. auris* and *C. auris* clades. We developed AS-PCR primers that yield successful PCR amplification of a *C. auris*-specific ITS2 amplicon but are insensitive to the species that *C. auris* is commonly misidentified as. For clade identification, we designed a duplex PCR that targets a region of ITS1 that is divergent for four of the five described clades (clades I and III have identical ITS sequences), while we used a clade III/V-specific gene cluster to discriminate clades I and III.

This assay can be used as a tool for epidemiological research and is, due to its low-cost and low-tech necessities, ideal for lower budget research settings including developing regions in which *C. auris* outbreaks have frequently occurred [14,41]. Moreover, clade-associated phenotypic traits such as virulence and drug resistance can make clade diagnostics an important tool for the clinic, by enabling a quick assessment of the risk for resistance-induced treatment failure or proneness to hospital outbreaks. Lastly, our clade diagnostic tool, along with a more in-depth analysis of the ITS region, can serve to identify potential new clades of *C. auris.*

## 2. Materials and Methods

### 2.1. Sequence Analysis for Species- and Clade-Specific Allele Selection

For the identification of *C. auris*, the ITS region was investigated for species-specific SNPs and/or indels. An alignment of 285–709 bp covering the ITS regions of 32 species was created in CLC Main Workbench v8.1 (Qiagen^®^, Hilden, Germany), by using TYPE/reference strain sequences from NCBI GenBank (ncbi.nlm.nih.gov/genbank/ (accessed on 8 February 2021)). Information about all sequences included in this alignment is listed in Table A1 (Appendix A). The selection of species was based on literature concerning the (mis)identification of *C. auris* [4,7,42,43,44] and includes most pathogenic *Candida* species [45]. The ITS alignment was manually searched for *C. auris-*specific regions to design a *C. auris* specific AS-PCR primer.

For the identification of *C. auris* clades, a 212–214 bp ITS alignment was created that contained 121 sequences: 96 typed *C. auris* strains from clade I, II, III, and IV as reported by Vatanshenassan et al. [31] (NCBI GenBank accessions MN242989–MN243084), two Iranian clade V strains [21,22] (NCBI GenBank accessions: MW019910.1 and MZ389242), and 23 *C. auris* strains from an in-house collection of isolates from all five clades. The latter consists of 15 clade I strains, three clade III strains, three clade IV strains, one clade II strain, and one clade V strain and includes at least one typed strain per clade for which whole-genome sequencing data are available (e.g., clade I: B8441, clade II: B11220, clade III: B11221, clade IV: B11244 [2,3,13,17], and clade V [12]: B18474/IFRC2087; see GenBank accessions PRJNA328792 and PRJNA541007, respectively). Strain information of the in-house collection is summarized in Table A2 (Appendix A). This alignment was manually searched for clade-specific regions to design AS-PCR primers. To illustrate clade divergence based on ITS, an UPGMA (unweighted pair group method with arithmetic mean) phylogenetic tree with Jukes–Cantor correction and 1000 bootstraps was created in CLC Main Workbench v8.1 (Qiagen^®^, Hilden, Germany). To differentiate between clade I and III isolates, which have identical ITS sequences, an l-rhamnose-1-dehydrogenase (*RHA1*) gene was targeted. *RHA1* is part of a rhamnose assimilation gene cluster present in clade III but not in clades I, II, and IV [46]. As data on the presence of this gene cluster were lacking for clade V, this sequence was extracted from the clade III *C. auris* isolate B11221 genome (accession PRJNA328792) and used as a query for an NCBI BLAST^®^ search against the SRA Illumina data of one Iranian clade V isolate B18474/IFRC2087 (SRX5786024) [12]. Reads were assembled in CLC Main Workbench v21.0.3 (Qiagen^®^, Hilden, Germany).

### 2.2. Strains and Media

To test the AS-PCR assay for *C. auris* identification, a panel of 15 species was used. This panel was composed of species for which the ITS region shows similarities to the *C. auris* ITS region and/or species that *C. auris* has been misidentified as [4]. This panel contained *C. auris* (B8441), *C. haemulonii* (CBS 5149), *C. duobushaemulonii* (CBS 7798), *C. pseudohaemulonii* (CBS 10004), *C. albicans* (SC 5314), *C. tropicalis* (CBS 94), *C. parapsilosis* (CBS 604), *C. glabrata* (CBS 138), *C. lusitaniae* (CBS 4413), *Candida orthopsilosis* (CBS 10906), *Candida metapsilosis* (CBS 10907), *C. sake* (CBS 159), *Candida dubliniensis* (CD36), *Candida krusei* (CBS 573), and *S. cerevisiae* (BY4741).

To test the AS-PCRs for clade identification, a panel of 23 *C. auris* strains was used. Strain origin, strain reference nr (if assigned), and strain clade as identified based on ITS sequencing, microsatellite typing, and/or whole-genome sequencing of each *C. auris* strain used in this project are listed in Table A2 (Appendix A).

All strains were grown on yeast peptone dextrose (YPD, 2% glucose) agar at 30 °C or 37 °C and stored in YPD liquid medium containing 25% glycerol at −80 °C.

### 2.3. DNA Extraction

Cells were dissolved in 400 μL of TE buffer (10 mM Tris (pH 1), 1% SDS, 1 mM EDTA, 100 mM NaCl, 2% Triton X-100) and 400 μL of PCI solution (phenol pH 6.7, chloroform, and isoamyl alcohol in a 25:24:1 ratio) and lysed by micro-bead shearing in a FastPrep^®^ homogenizer (30 s 6 m/s (MP biomedical™, Brussels, Belgium). The homogenate was centrifuged (10 min, 14,000 rpm), and 300 μL of the supernatant was mixed with 30 μL of sodium acetate (3 M, pH 5.2) and 900 μL of 100% ethanol. The extract was vortexed, cooled (−20 °C) for 30 min, and centrifuged (10 min, 14,000 rpm) at 4 °C for DNA precipitation. The DNA pellet was washed twice with 500 μL of 70% ethanol, dried at 37 °C, and dissolved in nuclease-free water. The concentration of DNA was measured using a NanoDrop™ Microvolume spectrophotometer (Thermo Scientific™, Waltham, MA, USA). 

### 2.4. PCR and Sequencing

The ITS region of all *C. auris* and non-*C. auris* strains used in this study (see Section 2.2) was sequenced to confirm targeted variable regions. The targeted sequences were amplified by PCR, using Q5^®^ High-Fidelity DNA polymerase (New England Biolabs Inc., Ipswich, MA, USA). The total reaction volume of 50 μL contained 500 ng of purified DNA, 5 μL of dNTPs (2.5 mM), 10 μL of 5× Q5 buffer, 0.5 μL of Q5 polymerase, and 0.4 μL of both universal fungal barcoding primers ITS1 and ITS4 (100 μM) (Table 1) [47]. The PCR program consisted of initial denaturation at 98 °C for 30 s, 30 cycles of 98 °C for 10 s, 59 °C for 25 s, and 72 °C for 30 s, and a final elongation step at 72 °C for 2 min in a Labcycler Basic thermocycler (Bioké, Leiden, The Netherlands). Correct amplification was verified by gel electrophoresis of 5 μL of the PCR product on a 1% agarose gel. Sanger sequencing (TubeSeq service) was performed by Eurofins (Nazareth, Belgium).

### 2.5. Allele-Specific Primer Design

**Figure 1:**
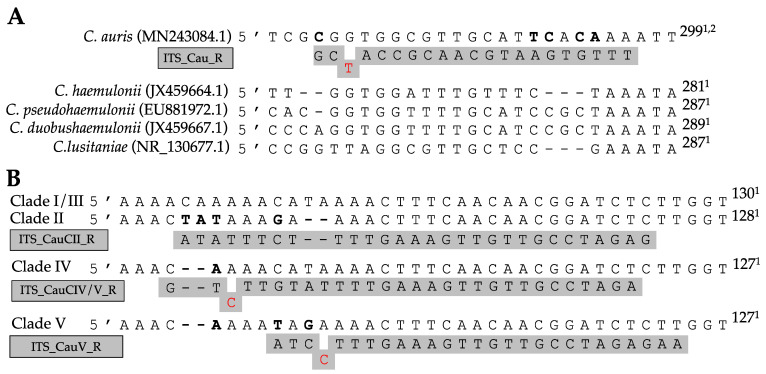
Allele-specific primer binding sites in the ITS region for the identification of *C. auris* (**A**) and *C. auris* clades (**B**). Specific polymorph nucleotides are shown in bold, primers are shown in gray, and mismatches contained within the primer are shown in red. ^1^ Nucleotide position from ITS1 forward primer. ^2^ Length based on ITS region of clade I.

Primers for AS-PCR were designed following the method of Liu et al. [34]. All allele-specific primers were designed in silico using CLC Main Workbench v8.1 (Qiagen^®^) and are listed in Table 1. To further increase the primer specificity, specific mismatches at the 3′ end of the allele-specific primer were implemented in several primers (see Figure 1). By including additional mismatches, the specificity of the primers for the right allele can be increased at higher annealing temperatures [34].

### 2.6. AS-PCR

All AS-PCRs were performed using *Taq* DNA polymerase with Standard *Taq* buffer (New England Biolabs Inc.). The total reaction volume of 20 μL contained 2 μL of purified DNA extract (10 ng/μL), 1.6 μL of dNTPs (2.5 mM), 2 μL of 10× Standard *Taq* buffer, 0.1 μL of *Taq* DNA polymerase, and the following primers: 0.1 µL of the ITS1 forward primer (100 μM) and the ITS_Cau_R reverse primer (100 µM) for species identification (simplex PCR) or 0.2 μL of the ITS1 forward primer (100 μM), 0.1 µL of the ITS_Cau_R reverse primer (100 µM), and 0.2 µL of the clade-specific reverse primer (100 µM) for clade identification (duplex PCR). The PCR program consisted of initial denaturation at 95 °C for 30 min, 35 cycles of 95 °C for 20 min, primer annealing at a primer specific temperature (Ta, see Table 1) for 30 min, and amplicon elongation for 30 min at 68 °C. The PCR was terminated by a final elongation at 68 °C for 5 min. All reactions were performed using a Labcycler Basic thermocycler (Bioké). Correct amplification was verified by 2% agarose gel electrophoresis of 10 μL of the PCR product. The specific annealing temperature of all AS-primers was identified by performing the same procedure as described above but using a 12-step temperature gradient from 50 °C to 70 °C and 60 °C to 80 °C as annealing temperature. From the window of specific amplification, one temperature was selected as annealing temperature (Ta).

## 3. Results

### 3.1. C. auris Identification

**Figure 2:**
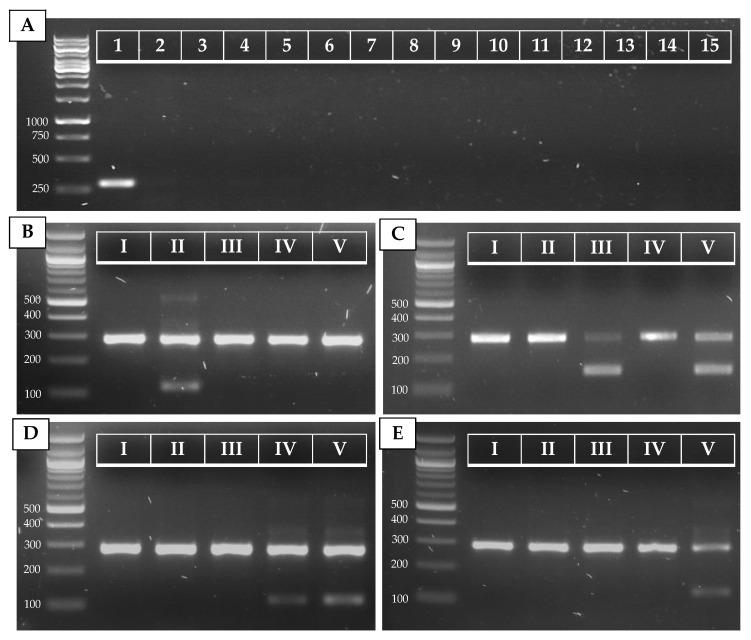
PCR results for *C. auris* (**A**) and *C. auris* clade (**B**–**E**) identification. (**A**) Simplex PCR of the ITS1–ITS_CauR primer pair at 78 °C Ta on 20 ng of purified DNA of *C. auris* (1), *C. haemulonii* (2), *C. duobushaemulonii* (3), *C. pseudohaemulonii* (4), *C. albicans* (5), *C. tropicalis* (6), *C. parapsilosis* (7), *C. glabrata* (8), *C. lusitaniae* (9), *C. orthopsilosis* (10), *C. metapsilosis* (11), *C. sake* (12), *C. dubliniensis* (13), *C. krusei* (14), and *S. cerevisiae* (15). (**B**–**E**) Duplex PCR of the ITS1–ITS_Cau_R and ITS1–ITS_CauCII_R (**B**), *RHA1*_CauCIII/V_F–*RHA1*_CauCIII/V_R (**C**), ITS1–ITS_CauCIV/V_R (**D**), and ITS1–ITS_CauCV_R (**E**) primer pairs at 68 °C Ta on 20 ng of purified DNA of *C. auris* B8441 (I), B11220 (II), B11221 (III), B11244 (IV), and B18474 (V), which are reference strains from clades I to V, respectively.

The alignment of 32 species showed great variation within the ITS regions. Figure 1A shows a fragment of the ITS2 region on which the *C. auris* specific reverse primer (ITS_Cau_R) was designed, amplifying a fragment of 296–293 bp when paired with the universal fungal barcoding forward primer ITS1 [47]. The allele-specific reverse primer contains a G–T mismatch at the third position of the 3′ end to increase specificity for *C. auris.* At an annealing temperature of 78 °C, *C. auris* but not *C. haemulonii, C. pseudohaemulonii, C. duobushaemulonii, C. albicans, C. glabrata, C. dubliniensis, C. tropicalis, C. parapsilosis, C. orthopsilosis, C. metapsilosis, C. lustianae, C. krusei, C. sake*, or *S. cerevisiae* DNA is amplified, as shown in Figure 2A. Sequencing the ITS region of our panel of 15 species confirmed the variability in the ITS2 region which was targeted. This region did not show variability between *C. auris* clades.

### 3.2. C. auris Clade Identification

**Figure A1:**
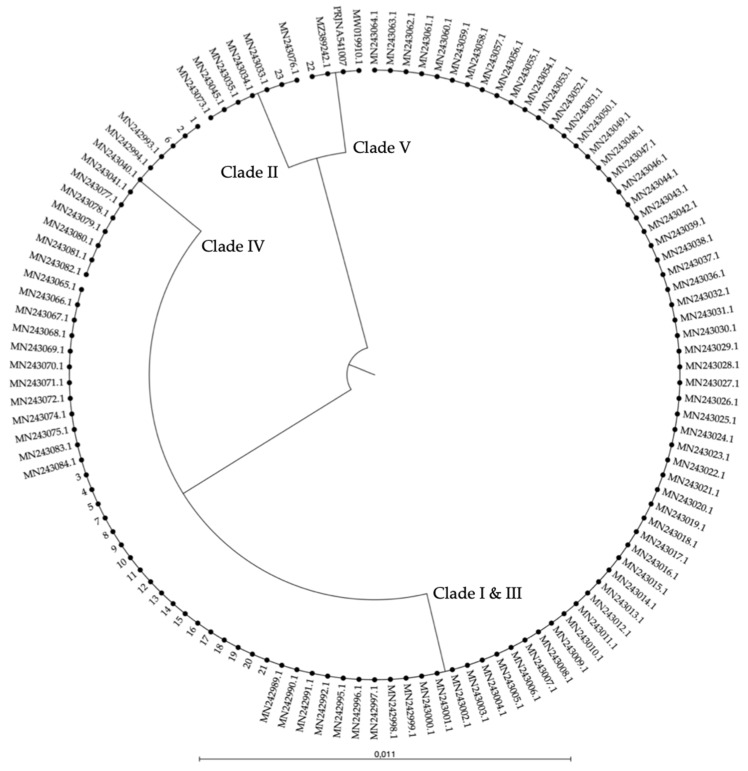
UPGMA dendrogram of a 248–251 bp ITS alignment of sequences of 121 *C. auris* strains: 96 typed strains from four clades [31] and three clade V strains [12,21,22], represented by their GenBank accessions, and 23 strains from our in-house collection, represented by the numbers 1 to 23 (see Table A2). Four ITS-based clusters are shown: clades I and III, clade II, clade IV, and clade V. The scale bar represents the percentage of nucleotide variation.

The ITS alignment of 121 *C. auris* strains from all five *C. auris* clades showed no intra-clade variability within the ITS region. Between clades, in both the ITS1 and the ITS2 regions, clade-specific polymorphisms were found. This resulted in four main ITS-based clusters (clades I and III, clade II, clade IV, and clade V), in a phylogenetic tree (Figure A1, Appendix A). A rhamnose assimilation gene cluster, reported to be present in clade III but deleted in clades I, II, and IV [46], was found to be present in the three clade V isolates that were investigated. Targeting this gene cluster and a clade V-specific ITS region enabled to discriminate clade III from clade I (Figure 2C,E).

Several strains from our in-house collection were typed on the basis of whole-genome sequencing data (e.g., strain 14 (B8441, clade I), strain 23 (B11220, clade II), strain 3 (B11221, clade III), strain 2 (B11245, clade IV), and strain 22 (B18474, clade V); see Table A2, Appendix A). We optimized and tested our AS-PCR primers using these strains and confirm correct clade identification (Figure 2B–E). Moreover, correct placement of the sequenced ITS region of these strains within the correct clade cluster reconfirmed clade phylogeny (Figure A1, Appendix A).

**Figure A2:**
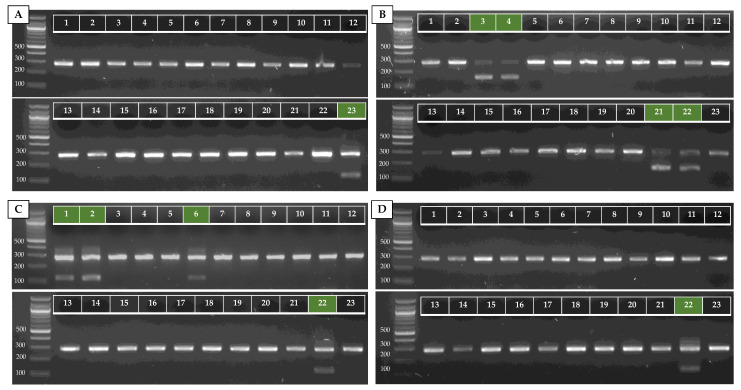
AS-PCR results of the four clade-specific PCRs for 23 strains of *C. auris* from our in-house collection, represented by the numbers 1–23 (see Table A2). Duplex PCRs of the ITS1–ITS_Cau_R and ITS1–ITS_CauCII_R (**A**), *RHA1*_CauCIII/V_F–RHA1_CauCIII/V_R (**B**), ITS1–ITS_CauCIV/V_R (**C**), and ITS1–ITS_CauCV_R (**D**) primer pairs at 68 °C Ta on 20 ng of purified DNA are shown. The green markings indicate where a positive result should appear.

To test our AS-PCR for clade identification, 23 *C. auris* strains (15 clade I strains, one clade II strain, three clade III strains, three clade IV strains, and one clade V strain) were screened. Information about these strains is summarized in Table A2 (Appendix A). The results of the four allele-specific multiplex PCRs in which the *C. auris*-specific amplicon (primers ITS1 and ITS_Cau_R) is duplexed with the clade II (primers ITS1 and ITS_CauCII_R)-, clade III and V (primers *RHA1*_CauCIII/V_F and *RHA1*_CauCIII/V_R)-, clade IV and V (primers ITS and ITS_CauCIV/V_R)-, and clade V (primers ITS1 and ITS_CauCV_R)-specific amplicons, is shown in Figure A2 (Appendix A). This shows that our multiplex AS-PCR assays for clade detection are 100% specific. Additionally, our multiplex design decreases the chance for false-negative results, as the *C. auris*-specific amplicon works as a positive control for successful PCR amplification.

## 4. Discussion

In this study, we show that combining the variability in the ITS region with the presence or absence of a rhamnose assimilation gene cluster can be used to identify *C. auris* and identify to which of the five main clades the *C. auris* strain belongs. The four AS-PCR assays for clade identification each consist of a duplex PCR reaction with a *C. auris*-specific amplicon and a clade-specific amplicon. This significantly reduces the chance of false-negative results in screening assays, as the *C. auris*-specific amplicon serves as an internal control. AS-PCR provides a rapid, low-cost, low-tech alternative to other clade identification methods such as (genome) sequencing or microsatellite typing. Nevertheless, we do recommend validating this diagnostic assay with sequencing and typed reference strains before use in screenings, as PCR-based methods can show variability due to technical discrepancies.

Molecular diagnostics are some of the most reliable identification methods for microorganisms. However, sequencing of molecular barcodes such as ITS requires time, specialized equipment, and analysis. Therefore, several sequencing-independent DNA-based diagnostic assays have been developed. These can be divided into three groups: end-point PCR assays (simplex or multiplex PCR and gel electrophoresis), quantitative PCR (qPCR) methods, and nonconventional detection methods such as loop-mediated isothermal amplification (LAMP) or T2 nuclear magnetic resonance measurement [9]. The method we report here belongs to the first group and only requires standard PCR reagents, a thermocycler, and gel electrophoresis setup. Such a method is ideally fit for low-budget research settings. Nevertheless, our method has some potential disadvantages. It is, like most diagnostic methods, a culture-dependent assay. Additionally, no non-*auris Candida* species can be detected, and typing is limited to the five currently described clades. Nevertheless, AS-PCR can be optimized as a qPCR assay for culture-independent diagnosis, as reported with other qPCR methods for detection in clinical samples [8], and other species-specific primers can be designed.

We used the ITS region for species identification as this is the primary fungal barcoding marker, proposed by the Fungal Barcoding Consortium [48]. The ITS region consists of two spacer sequences surrounding the 5.8S rRNA gene in the ribosomal cistron and allows successful identification of a broad range of fungi with a clearly defined barcoding gap between inter- and intraspecific variation [48]. Moreover, single-copy protein-coding regions often show lower PCR amplification and sequencing success compared to the multicopy ITS region, which yields a PCR amplification success of 100% for Saccharomycotina, the subphylum to which *Candida* species belong [48]. ITS sequencing analyses [4], as well as ITS-based diagnostic PCR assays [44,49,50], have been widely reported for *C. auris* identification. In addition to species-level identification, ITS sequencing has been used for typing *C.* *auris* and other fungal species in the past [31,48,51]. Here, we show that the ITS sequence harbors sufficient interclade diversity to discriminate four out of five clades. An l-rhamnose gene cluster was used to discriminate clade III from clade I, as they share the same ITS sequence. The l-rhamnose gene cluster contains seven genes (*RHA1*, *LRA1*, *LRA2*, *LRA3*, two copies of *TRC1*, and an *MFS* transporter) which are absent in clade I, II, and IV isolates but present in clade III [46]. This pattern was discovered by testing an updated Vitek 2 yeast identification system, in which all clade III isolates but hardly any other *C. auris* isolates or *C. haemulonii* showed the ability to assimilate l-rhamnose [46]. This phenotype has not been validated for clade V isolates, but here we show that the l-rhamnose gene cluster is present in three clade V strains, including the type specimen [12].

Clade typing can be of significant epidemiological value, as it provides information on the origin of a strain and can help to monitor nosocomial transmission. Moreover, clade diagnosis can have a clinical value, as clade-associated virulence and resistance implications have been reported [2,3,12,17,20,21,22,23,24,25,26,27,30,52]. Identification of the clade to which *C. auris* isolates could belong in an outbreak might, thus, have implications for the choice of treatment and the use of infection control and prevention measures. In scientific research, clade identification is also essential. Molecular and pharmaceutical research of *C. auris* implies the use of isolates from different clades due to the outspoken phenotypic difference between clades and the possible implications for scientific conclusions on a species level [2,3,17,20,21,22,23,24,25,26,27,30,52]. Another useful purpose of screening isolates with this diagnostic AS-PCR assay is the potential to identify new clades. When the patterns of clade-specific PCRs differ from an expected outcome, this could be an indication of a novel, undescribed clade. Whole-genome sequencing and microsatellite typing will provide more in-depth insight in such circumstances. Detection of new clades could have a profound impact on our understanding of the emergence and epidemiology of this novel fungal pathogen.

In conclusion, we provide a molecular diagnostic assay to identify *C. auris* and the five currently described *C. auris* clades, which can be used for epidemiological, pharmaceutical, clinical, and molecular research. The low-cost and low-tech necessities and fast readout make it a convenient tool, ideal for low-budget research settings. As the number of reported *C. auris* cases and outbreaks is still on the rise, the use and development of up-to-date, reliable identification and typing tools is essential.

## Figures and Tables

**Table 1:** Primers used in this study. The specific annealing temperature (Ta) is given in °C.

Primer Name	Sequence	Use (Ta)	Target	Reference
ITS1	TCCGTAGGTGAACCTGCGG	Sequencing (59) AS-PCR (68 or 78) ^1^	Fungi	[47]
ITS4	TCCTCCGCTTATTGATATGC	Sequencing (60)	Fungi	[47]
ITS_Cau_R	TTTGTGAATGCAACGCCATCG	AS-PCR (78)	*C. auris*	This study
ITS_CauCII_R	GAGATCCGTTGTTGAAAGTTTTCTTTATA	AS-PCR (68)	Clade II	This study
*RHA1*_CauCIII/V_F	TTGCGGTTGAAATGGGTGCT	AS-PCR (68)	Clade III/V	This study
*RHA1*_CauCIII/V_R	TGGCATGTTTCCGGCTTAGA	AS-PCR (68)	Clade III/V	This study
ITS_CauCIV/V_R	AGATCCGTTGTTGAAAGTTTTATGTTCTG	AS-PCR (68)	Clade IV/V	This study
ITS_CauCV_R	AAGAGATCCGTTGTTGAAAGTTTCCTA	AS-PCR (68)	Clade V	This study

^1^ A Ta of 68 °C was used for multiplex AS-PCR to identify clades, while a Ta of 78 °C was used for *C. auris* species identification.

## Data Availability

All original data are available from the authors. No large datasets were generated.

## Appendix A

**Table A1:** All species included in the alignment to identify a *C. auris*-specific ITS region for the design of primer ITS_Cau_R. The species and strain names are given, along with the ITS sequence NCBI GenBank accession and the size of the sequence in the alignment after trimming.

Name	NCBI Accession Nr.	Strain	Size (bp)
*Candida auris*	NR_154998.1	CBS 10913	342
*Candida haemulonii*	JX459664.1	CBS 6590	479
*Candida haemulonii* var. *vulnera*	JX459687.1	CNMCL7256	322
*Candida pseudohaemulonii*	EU881972.1	C4368	327
*Candida duobushaemulonii*	JX459667.1	CBS 7799	487
*Candida albicans*	NR_125332.1	CBS 562	462
*Candida tropicalis*	NR_111250.1	CBS 94	454
*Candida lusitaniae*	NR_130677.1	CBS 6936	307
*Candida glabrata*	NR_130691.1	NRRL Y-65	655
*Candida dubliniensis*	NR_119386.1	CBS 7987	460
*Candida parapsilosis*	NR_130673.1	ATCC 22019	461
*Candida orthopsilosis*	NR_130661.1	ATCC 96139	436
*Candida metapsilosis*	NR_165186.1	CBS 10907	472
*Candida sake*	NR_151807.1	CBS 159	432
*Candida inconspicua*	NR_111116.1	CBS 180	379
*Candida castellii*	NR_154958.1	CBS 4332	628
*Candida bracarensis*	NR_136973.1	CBS 10154	704
*Candida pararugosa*	MF797780.1	S200	324
*Candida guilliermondii (Meyerozyma guillermondii)*	NR_111247.1	CBS 2030	532
*Candida krusei (Pichia kudriavzevii)*	NR_131315.1	ATCC 6258	435
*Candida famata (Debaryomyces hansenii)*	NR_120016.1	JCM 1990	580
*Candida kefyr (Kluyveromyces marxianus)*	NR_111251.1	CBS 712	628
*Candida norvegensis (Pichia norvegensis)*	MH396411.1	URM 7762	434
*Lodderomyces elongisporus*	NR_111593.1	ATCC 11503	496
*Yarrowia lipolytica*	NR_111212.1	CBS 6124	285
*Nakaseomyces delphensis*	NR_167682.1	CBS 2170	709
*Nakaseomyces bacillisporus*	NR_138202.1	NRRL Y-17846	662
*Kodameae ohmeri*	NR_121464.1	CBS 5367	348
*Diutina catenulata*	NR_077200.1	CBS 565	343
*Rhodotorula mucilaginosa (R. rubra)*	NR_073296.1	CBS 316	557
*Rhodotorula glutinis*	MH665424.1	R.g0726	582
*Saccharomyces cerevisiae*	NR_111007.1	CBS 1171	613

**Table A2:** All *C. auris* strains used in this study to validate clade identification AS-PCRs. The strain name (if assigned), origin, clade, and reference to typing literature of the reference strains (bold) are indicated. Clade identification is based on ITS sequencing, microsatellite typing, and/or whole-genome sequencing.

Nr.	Strain Name	Origin	Clade	Typing Reference
1	B11244	Venezuela	IV	[2,13,17]
2	B11245	Venezuela	IV	
3	B11221	South Africa	III	[2,3,13,17]
4	B11222	South Africa	III	
5	CDC_390	India	I	
6	-	Colombia	IV	
7	B11098	Pakistan	I	
8	B11203	India	I	
9	-	India	I	
10	-	India	I	
11	-	India	I	
12	-	India	I	
13	VPCI 714/P/14	India	I	
14	B8441	Pakistan	I	[2,3,13,17]
15	317052804	Oman	I	
16	VPCI 514/P/14	India	I	
17	VPCI 1133/P/13	India	I	
18	B11109	India	I	
19	VPCI 1131/P/13	India	I	
20	OS299	Belgium	I	
21	MRU224	South Africa	III	
22	IFRC2087/B18474	Iran	V	[12,17]
23	B11220	Japan	II	[2,3,13,17]
